# The Jew in Medicine1Read before the Buffalo Academy of Medicine, Medical Section, December 14, 1897.

**Published:** 1898-02

**Authors:** Julius Ullman

**Affiliations:** Attending physician German Hospital Dispensary


BUFFALO MEDICAL JOURNAL.

Vol. XXXVII. FEBRUARY, 1898. No. 7.

### Original Communications.

### THE JEW IN MEDICINE.[1]

By JULIUS ULLMAN, M. D.,

Attending physician German Hospital Dispensary.

THE Jew, in the centuries which have passed, has been the target for persecutions and massacres and a scape-goat against whom was directed the contempt of the semi-barbarous nations; but though the hand of his oppressor may have dwarfed his body and painted, with too vivid a coloring, pain and suffering upon his countenance, his mind and spiritual self have ever remained his own and have, in part, given to posterity that knowledge in science, philosophy and the arts which it now possesses.

In no field of the Jew's activity, unless it be in the perpetuation of the divine laws, has more been accomplished by him, directly and indirectly, than in medicine, and it is in this channel of work, so well performed in the past, to which the writer would refer.

The ancient Jews were but a part of the Semitic nations—Phœnicians, Assyrians and Babylonians—all of whom had perhaps a higher degree of civilisation than they, and hence the Jews were influenced by them because of their involuntary exile among them. Unlike these nations, the Jews were not believers in mythology, but were monotheists and regarded Jehovah not only as their one and only God, but as their physician, for the bible reads: "I, Jehovah, am thy physician."

Jehovah was regarded as the great cause of disease, for He punished with disease the transgressors of His commandments. Deuteronomy, xxviii.–21, reads: "The Lord shall make the pestilence cleave unto thee, until he have consumed thee from off the land, whither thou goest to possess it." Again, the bible reads: "The Lord shall smite thee with the botch of Egypt, and with the

1. Read before the Buffalo Academy of Medicine, Medical Section, December 14, 1897.

emerods, and with scurvy, and with the itch whereof thou canst not be healed."

It is inferred from the above quotations that the Hebrew was taught the theological doctrine of the cause of disease, but, unlike other creeds, did not believe in the theological doctrine of the cure of disease.

Moses is the father of medical police and public hygiene. The Mosaic laws, in their beneficent wisdom, are instruments by which he influenced the Israelites.

Circumcision was introduced as a religious ritual, and of its import, as to cleanliness and immunity to disease, but little need be said here.

The terming of a woman during the few days following her menstrual period as unclean and unfit for cohabitation is mentioned, and Moses here displayed his wisdom, not only as a hygienist, but as a statesman and sociologist. He did not desire too great a fruitfulness, especially as it was said, and is known, that Jewish women bore children oftener and more easily than did the Egyptians.

The laws, among other things, forbid the marriage of kindred, speak of the situation of cemeteries, time of burial, isolation of the sick (certainly a forerunner to the study of infection, the germ theory of disease,) and the use of vessels employed by the latter. There are strict regulations as to the kind and preparation of food and precepts as to the admissible animals. Swine were especially not allowed. This was due to the prevalence at that time of a disease simulating trichinæ spiralis poisoning. It is strange if something as to the relationship between the cause and the effect of the disease should not have been known to Moses.

The animals for slaughter are inspected by especially trained observers, called chohets, and if the meat is diseased it is unclean.

In this law lies the truth that pulmonary tuberculosis is very rarely seen, comparatively, among the Jews. The records of the Jewish Hospital, of New York, according to Remondino, give, out of 28,750 persons admitted, only 44.57 per 1,000 of its admissions as being due to phthisis ; while those of the Roosevelt Hospital, out of 25,583 admissions, give, as per 1,000, 67.93.

Rev. Dr. Louis Grossman, in an article on the medicine among the Jews, says :

One provision of the Mosaic dispensation is worthy of a special remark. Moses provides that a man who has recently married a woman shall not go to war within a year after his marriage. Moses knew that soldiery constituted the most virile part of the people and he wished to conserve the sturdy quality of the reproductive capacities. What wars have done toward depriving the nations of their most healthful stock, and of the vital germs of other possible lives, we cannot surmise.

From the foregoing it is seen that, from earliest times, the Jews were taught that the observance of certain ordinances were both conducive to health and the prevention of disease. This was equivalent to the teaching that disease originated from physical causes and that physical means were to be used to combat disease. Ecclesiastes xxxviii. reads : "When thou art sick call upon God and bring a physician, for a prudent man scorneth not the remedies of the earth."

There was a practice in vogue among the early Hebrews of exposing the sick in public places, so that they might receive the benefit of those who had experience in a similar case before. So, from religion and custom the medicine of the Hebrew has received its foundation of intelligent philosophy which was to carry it in its purity through all ages free from magic, superstition and imposture.

The Levites, in their priestly calling, are to be regarded as the first body of physicians, and of their private practice but little is known. Their office may be likened to that of a quarantine officer of today. They investigated the purity of men and women and examined suspected cases of leprosy. When pronounced unclean, the patient was sent without the camp and dare not return until he had performed ablution and was pronounced clean again.

King Solomon and the prophets also acted in the capacity of physicians. The Talmud opened a new era in medicine. After the destruction of Jerusalem and its temple by Titus, 70–79, and the simultaneous downfall of the Jewish schools, colleges were erected in distant parts for the preservation of the pure faith. These institutions were presided over by eminent rabbis who were versed in medicine. Rabbi Gamaliel taught at Jabuet, near Jerusalem ; Rabbis Simeon and Jehuda Hakkadesch at Tiberias ; Rabbi Samuel at Nahardea, in Mesopotamia ; Rabbi Abba Arecha at Nechesja, on the Euphrates ; Rabbi Judas at Alexandria, and Tobias, of Modain, at Jerusalem.

From a modern standpoint, the Talmud is to be considered as an encyclopedia. It consists of the interpretations, traditions and decretals of the rabbis in the most diverse directions and on the most varied topics, philosophical, religious, medical and legal. The medicine of the Talmud was very crude. Diseases were named as to the organs affected, such as heart disease and intestinal diseases. Sometimes they were named from the principal symptoms, as jaundice, dropsy and the like. Surgically, it considers dislocations, contusions, trauma of the vertebræ, perforations of stomach, esophagus or intestines. It was considered dangerous to operate with iron instruments; wooden were employed. Cesarian sections were performed. Diagnosis was purely empirical. There were critical signs for prognosis, such as perspiration, sneezing, dreams, pollutions. Anatomy was somewhat understood, for, according to the Talmud, the human body consisted of 248 bones. It is related (Talmudic Miscellany, Husham, 1880; Trübner's Oriental series) of Rabbi Ishmael's disciples that they dissected a low woman, who had been condemned by the government, and that they found 252 bones.

Of greatest importance is the Talmud as a work of hygiene. It treats of uncleanliness, with many sanitary rules and regulations. It treats of plagues, contagions, disorders, leprosy and methods of isolation. It treats of houses as well as tents, with special reference to the contaminating presence of the corpse. It prescribes the immersion bath and especially gives rules as to the cleansing of hands.

The era of the Talmud was the first five centuries. With the medicine of the Talmud terminated medicine as treated in special Jewish works, for history seems to have ignored the Jews as a nation so soon as they quit wielding the sword, and the Jews lost their individuality in literature, but became the teachers and translators for other nations.

During the third and fourth centuries the Jews were the victims of persecutions by the Persians (and Bishop Cyrellin, of Alexandria), but with the birth of Abul Kasem Mohammed, 571 A. D., the Arabian empire was born with a new religion and a thirst for higher knowledge. The Arabs became possessors of Syria, Palestine and Egypt and became acquainted with Greek culture. They were the friends of culture and the lovers of the learned, and the Jews found here a friendly home among the Arabs, for the Arabic language was closely allied to the Hebrew, and Hebrew literature was admirably adapted to the taste of the Arabian. The Jews became the greatest teachers in the Arabian schools situated at Basra, Bagdad and Kufa.

During the eighth and ninth century it was not infrequent to find that the Jews were ordinary, or body, physician to the caliphs. The Jewish physicians reached such repute in Arabia that they invoked the jealousy of the Arabians, and Caliph Montawakkel decreed that the Jewish students should be taught only in the Arabian and Syrian language, not in the Hebrew.

One of the greatest physicians of this time was Isaak Ben Soleiman, 830 to 940. He wrote on fevers, dietetic subjects and deportment and conduct of the physician. He introduced senna, a drug now universally used. He was ordinary physician to the sultan of Morocco, Abu Mohammed el Mahdi. He died a centenarian, beloved by all, and so important were his writings that his works were still published in Venice in 1515, six centuries after his death.

The Jews were a great maritime race. They were the possessors of fleets which were to be seen in all the known parts, so that by their contact with the foreign nations they inculcated the influence of their ancient wisdom and in return learned the foreign languages, thereby becoming the great linguists of those ancient days. It therefore became possible for the Jew to introduce into Western Europe a portion of the science of the ancients.

When science was renewed it began in the foundations laid down by the Greeks. Adam, in his civilisation during the middle ages, writes: "In every line the first step was to find out what the ancients had known and then to begin a new progress from the point which they had reached." The first medical lectures then were but comments on the Greek text. Here, again, the Jewish physicians showed their ability because of their aptitude as translators and interpreters of the text.

Salerno, south of Naples, and Montpellier, in France, were the renowned schools of learning. Here not only was divinity and philosophy taught, but medicine as well. In Salerno, Elisus, a Jew, was one of a trio of learned men who gained such a reputation for the school, and Copho, one of its pupils, whom Häser mentions as one of the greatest physicians and writers of his time, was a Jew. At Montpellier the Jewish physician, Jakub Ben Makir, was rector of the university and to this day its school of medicine remains as her chief ornament.

It may, however, be said that before the rise of the schools of Salerno and Montpellier the only physicians throughout the then known world were Jews, and though they were afterward joined by the Arabs in Spain they again became, at the expulsion of the latter from the country, the sole representatives of the medical sciences. Because of their skill the Jewish physicians occupied important offices of trust in the government, so we find Hasdai Ben Shahroot the physician and prime minister to Abdahrahman III. of Cordova. Isaac Arbarbanel, minister of France under Ferdinand and Isabella, is said to have been educated as a physician.

In those dark and middle ages, when men reasoned nothing from a physical basis, but attributed all phenomena to a supernatural agency, either heavenly or diabolical, the old maxim that "in ignorance there is bliss for it is folly to be wise" was so true. If the renown as physicians and their medical skill forced the Jews into places of trust and on the pinacles of fame, it also exposed them to inevitable dangers. If successful the physician was liable to a suspicion of sorcery and unlawful dealing. Knowledge and magic were so closely allied in the popular mind, that a wonderful cure wrought by a Jew could not be wrought by science, still less by divine aid, therefore must be wrought by diabolic aid. If the Jewish physician were unsuccessful, the dying patient must have been the victim not of an incurable malady or even ignorance. The Jewish physician must knowingly have administered poison, as was charged against Zedekiah, the court physician of Charles the Bald, 798 to 814.

The church held science as the seed of the devil. The monk and the priest were prohibited from studying medicine. In order that religion might control the body as well as the mind, science was allowed to slumber. Still the priests disported themselves and even received the title of physicians. Medical practice became debased, as may be inferred from the fact that prayer, the imposition of hands, exorcism, rings, sacred symbols, holy oils, bones, rags, sacred relics, conjurations, crossings, and the like, were openly employed by the Catholic clergy. Is it to be wondered that with such medical practices that there were great plagues and epidemics?

The Jews, because of their knowledge of the Pentateuch and their study of the Talmud, were exempt from plagues and epidemics. Dr. B. W. Richard, on Relation to race to disease, states that from epidemics the Jews have often escaped as if they possessed a charmed life. Because the Jews were exempt from these plagues and epidemics they were said to be in league with the devil and to have poisoned the wells, and as a result, Remondino states, that the rational and law of nature observing Jew could neither be seen or heard in his own defense. The cry of Hep, Hep, (Hep, Hep, three initials of three words *Hirrusalem est perdita*, according to Isador Loeb—Heb, Heb, Stop! Hold him!) was raised, and it spread, gained in intensity, and such insane torture as the popular fury could suggest were the human manifestations with which a Christian people visited their Jewish brothers, whose only sin consisted in worshiping the God of their fathers and in strictly observing His laws and commandments.

Among the reasons given for the expulsion of the Jews from Spain, in 1492, was the crucifixion at different times and the poisoning of patients by Jewish physicians.

It was in this same Spain that the golden age of the Jewish physician shone with the mightiest and most enduring splendor. This period extended until the end of the X. century. While Christian Europe lay in darkness, Mohammedan Cordova might be considered the center of civilisation, arts and letters, and Millman, in history of the Jews, states that the Jews, under the employment of equal rights and privileges, rivaled their masters, or rather their compatriots, in their advancement to wealth, splendor and cultivation.

It was at the end of this period that two great Jewish physicians lived. Abernan Avenzoar, whose father, cousin and grandfather were physicians, was born about 1070 at Pentaflor, near Seville, in Spain. He was the most noted physician of his time in Spain and Africa. He died in 1162, honored as the wise and illustrious. He practised surgery, although in his time it was considered as a disgrace. He performed experimental operations; the first total extirpation of the uterus is attributed to him and therapeutically he recommended the milk cure in consumption. He wrote eight works, one of which was a treatise on fevers, De febribus, which was printed in Venice as late as 1497.

About the same time there lived the greatest of physicians among the Jews, Rabbi Ben Moses Maimon, called Maimonides. He was the speculative parent of Spinoza and Mendellsohn. He was born in Cordova in 1135. In his early manhood he was a judge, but, unfortunately, he was the victim of persecutions against his people by the Mohammedans, and seeking refuge in various countries at length settled in Feshat, opposite Cairo. Here the Sultan Salaheddin, in Jossuf Ben Ajub, took the learned Jew under his protection and the fame of Maimonides, as one of the most profound philosophers and learned physicians among the most enlightened Jews and Arabians, grew to its height. At early dawn he used to cross to Cairo, where he was court physician, and on his return there were great crowds who came to consult him on questions medical, philosophical and religious. He writes his friend Samuel ib Tibbon, on leaving Cairo and arriving at his home in Feshat, "I find the ante-chambers filled with people, both Jew and Gentile, nobles and common people, judges and bailiffs, friends and foes; a mixed multitude, who await the time of my return. Patients go in and out until nightfall and sometimes even until two hours in the night. I converse with them and prescribe for them while lying down from sheer fatigue, and when night comes I am exhausted so that I can hardly speak." Häser mentions seven of his works: 1. *Trœratus de regemimi Sanitatis.* 2. *Aphorismi medici*, out of Galen's writings. 3. *Commentarius in Hippocrati Aphorusimus.* 4. *Libii niventi*, Hebrew medical and moral contents. 5. *Tractu de hœmorrhoidibus.* 6. *Tractus de cura corum qui a Venenatus animalibus punetisunt.* 7. *De causis et medicis morborum.*

From the XIII. to the XVI. centuries the Jews played an important rôle in medicine in Spain. Their great effort enabled the translation of the best Hebrew, Arabic and Greek literature, into a condensation of Latin works.

After the edict expelling the Jews from Spain, among whom the Jewish physicians were also included, they sought refuge in Italy, France, Turkey and the Netherlands. What a political suicide this insane act on the part of Spain seems to us so far removed from those miserable days of the inquisition; still it was but the emancipation of science and free thought from the bonds of theological dogmas and doctrines. Spain, having lost in the Jew the elements of its industrial success and scientific activity, has shrunken from the magnificent splendor of its former self until now it is barely able to hold with its tyrannical hand the few colonies, striving for independence, remaining to it of all its former possessions.

On the other hand, as the seed scattered by the wind and falling on fertile soil sprouts forth, and grows with renewed vigor, so Jewish thought in more encouraging environments again asserted itself and we hear of the Jewish physicians of Spanish or Portuguese descent doing noble work in foreign countries for the advancement of medicine.

In Italy the Jewish physicians were always much respected for their learning. I have noted on a previous page that one of the founders of the great school of Salerno was a Jew. As early as the XIII. century Häser mentions the Jew Ferraguth (Ferragius of Salerno) as the most important translator of Arabic writings into Latin. There were many Jewish physicians established there before the influx from Spain.

In many cases they enjoyed the friendship of popes and princes. Popes Julius II. and III., Leo X., Clemmens VII., and Paul III., had Jewish physicians. But often they were in attendance on popes or potentates who wrote bulls with restrictions, and oppressions against their people, and, in several instances, they were denied the practice of their chosen and honored profession among the gentiles. This was greatly due to the jealousy of fanatical monks; one in particular, Bernardin, of Sienna, traveled from city to city and injected wherever he could his poisonous and virulent thoughts against the Jewish physician. No misrepresentations were base enough, nor were lies small enough to portray before the eyes of the people pictures of horror and terror so great that they feared to call in a Jewish physician to the bedside of a dear one. One of the arguments used was that it was better to die than thank one's life to a Jewish physician.

The names of those in Italy who did so much for medicine are many. It is not my purpose to go too much into detail, but to present a sketch of the standing and work of the Jewish physician in those days of the middle ages. A few names may, however, be mentioned of Italian physicians worthy of note. The family of Porta De Leon are bright names in the history of medicine. Benjamin Porta De Leon was physician to King Ferdinand, of Naples. His son, Elieser, strove hard to walk in the footsteps of his illustrious father; a grandson, David Porta De Leon, who received a medical diploma at the University of Padua and who practised in Muatua, and his own son in turn, Abraham Porta De Leon, born in 1542, at first a rabbi, then theologian, but later one of the greatest physicians in Italy. He was one of the first who studied gold for its therapeutic value.

The Spaniard, Jehuda Abarbarnel, son of the finance minister under Ferdinand and Isabella, whose brother Joseph was also a physician, was a medical writer and poet of international reputation. Jacob Montino was also a celebrated physician of the period. He translated Averroes work into Latin. Besides these illustrious men there lived many others in Italy, one of whom deserves especial mention. Bonet De Lates was born in 1498. He went to Rome, and after devoting many useful years to the study of astronomy, became body physician to Pope Leo X. Bonet De Lates is especially remembered by the Jews for his kind offices in saving the Hebrew prayer books from destruction, for in 1509 there lived in Cologne one Pfefferkorn, who sought notoriety by attempting to confiscate and destroy the Jewish prayer books. He thought that by this means Judaism might be exterminated for all time. For many years there waged a war of the pen between Pfefferkorn and Reuchlin, a learned man and translator, who referred the argument to Pope Leo X. Bonet De Lates used his influence with the Pope, and because of his advice, Pfefferkorn, the fanatic, was defeated.

In France the Jew in his medical practice underwent much of the dreary life history of his contemporaries in other lands of which we have spoken. The most renowned physicians were those who came to it as weary emigrants from Spain and Portugal in order that they might worship, without fear of the inquisition, the religion of their forefathers. In the XVI. century there lived Ely Montalto, physician to King Henry IV. Orabio De Castro lived in Toulouse as a new Christian (*Schein-Christ*). He earned the title of royal physician, but soon sickening of the hypocrisy under which he lived, wandered to Amsterdam, where he asserted his religion and enjoyed a lucrative practice. John Baptiste of Silva was born in Bordeaux and was a descendant of a Portuguese family. He studied in Montpellier and was physician to King Louis XV. and to the German Karl, duke of Bavaria. Fonseca, also a Portuguese, was a noted physician and friend of Voltaire.

As we will see later in its treatment of the Jew, though France was many times unjust it was the first nation to grant to him a most humane treatment. It opened to him its arms and said to him, "Thou art also my son."

In Turkey and the Netherlands there also lived many illustrious Jewish physicians whose ability and renown was no less than those of Spain, Italy and France. In the Netherlands Spinoza (1632–1671), whose father was a physician, by his philosophical writings did much toward removing the hindrances of free thought.

The Jews, as physicians, in Germany did less than in other countries. The hatred of the Jews was especially virulent there. Every crusade was accompanied by malicious accusation in order to excuse designs of pillage and plunder. The rabble were always easily influenced for evil acts. Their anger and wrath once kindled the unhappy Jew became the victim of fire, sword and water.

The Jewish inhabitants were, like those of France, in a state of serfdom, dwelling in miserable huts, cut off from God's gifts to His children, sunlight and fresh air. They were huddled in the miserable ghettos (Judengasse), whence they could come out only at certain hours. Being recognised by a special sign of their servitude (called Judenfleck) they became victims of abuse from their fellowmen.

It was in such environments that the Jewish physician was to practise his profession. Many times they could minister only to their co-religionists. Their outward degradation worked inward on their minds, because confined to base and sordid occupations they contracted the thoughts and feelings to their station, and as the despised will become despicable, so the Jew, who had before written commentaries, cut off from the privileges of education and practice, often became a charlatan, wandering from village to village, but never remaining long enough to see a cure effected.

Despite these persecutions there are recorded many illustrious Jewish physicians, such, for instance, as Zedekiah. They lived in Nuremberg, Frankfurt, Berlin and Hamburg, and often received dispensations to live and practise without the pale of the Judengasse.

It was the philosopher, Mendellsohn, who paved the way for the development which physicians of the Jewish religion have made today. He had the Jew forget the Jewish jargon (a mixture of Hebrew and German), and learn the literature of the day at the expense of their Talmudic teachings. It prepared the way for that which followed, for when the French populace saw how the American colonists had founded their noble government, with its freedom of thought and religion, there was born in the cradle of their discontent the French Revolution, a war of the masses against the classes, full of such terrible historic scenes, but the people won their cry of liberty, equality and fraternity, and the Jew, for the first time in centuries, was recognised as a citizen and an equal. Napoleon I., in 1806, firmly established this right.

Since this time other civilised continental countries have also given to the Jew equal citizenship, and have opened to him schools and universities, so that Jewish medicine has, in our time, lost its identity, for it is a part of the medical history of his adopted fatherland; so that when speaking of his works today we must speak of him, not as a Solis-Cohen, Jacobi, Forchheimer, or Einhorn, a Jew, but as an American; not as a Bamberger, Kaposi, Neumann, or Politzer, a Jew, but as the Austrian physician ; not of Israel, Klemperer, Senator, Oppenheim, Ebstein, or Mendelas Jewish, but German physicians.

REFERENCES.—Baas, History of Medicine ; Häser, Geschichte der Medicin ; Renon-ardo, History of Medicine ; Roswell Park, An Epitomé of the History of Medicine ; LeRoy Beallean, Israel Among the Nations ; Dr. Richard Landau, Geschichte der Judischen Arzte ; Milman, History of the Jews ; Remondino, Circumcision.

400 FRANKLIN STREET.

